# How can we successfully recruit depressed people? Lessons learned in recruiting depressed participants to a multi-site trial of a brief depression intervention (the ‘CLASSIC’ trial)

**DOI:** 10.1186/s13063-018-3033-5

**Published:** 2019-02-13

**Authors:** June S. L. Brown, Caroline Murphy, Joanna Kelly, Kimberley Goldsmith

**Affiliations:** 1 Psychology Department (P077), Institute of Psychiatry, Psychology and Neuroscience, King’s College London, De Crespigny Park, London, SE5 8AF UK; 2 King’s Clinical Trials Unit (PO64), Institute of Psychiatry, Psychology and Neuroscience, King’s College London, De Crespigny Park, London, SE5 8AF UK; 3 Biostatistics & Health Informatics Department (PO20), Institute of Psychiatry, Psychology and Neuroscience, King’s College London, De Crespigny Park, London, SE5 8AF UK

**Keywords:** Depression, Recruitment, Self-referral, Psychological treatment, Self-confidence, Self-esteem, Non-diagnostic label

## Abstract

**Background:**

There are enormous problems in recruiting depressed people into randomised controlled trials (RCTs), with numerous studies consistently failing to recruit to target (Sully et al., Trials 14:166, 2013). Given the high prevalence of—and disability associated with—depression, it is important to find ways of effectively recruiting to RCTs evaluating interventions. This study aimed to test the feasibility of using a self-referral system to recruit to a brief intervention in a multi-site trial, the CLASSIC trial of self-confidence workshops for depression. In that trial, participants referred themselves to a depression intervention with a positive non-diagnostic title of ‘self-confidence’, given the close relationship of depression and self-esteem (Horrell et al., Br J Psychiatry 204:222–233, 2014).

**Method:**

We analysed uptake and retention rates by recruitment to the study, attendance at the workshops and follow-up rates. However, because of the rapid rate of recruitment, we decided to pause the trial and revise our original single-site research protocol in months 5–6. We report findings under three main headings: recruitment rates for the 12 months of the project before and after the pause; data regarding attendance at the workshops before and after the pause; and the follow-up rates before and after the pause.

**Results:**

We recruited 459 participants within 12 months, representing 38 participants recruited per month. Improved uptake of the intervention and retention after the development of multi-site research protocols are reported.

**Discussion:**

Based on previous evidence from RCT recruitment among depressed participants, our recruitment rate demonstrates that a self-referral system using a non-diagnostic title of self-confidence is a successful recruitment method. The implications of rapid recruitment using a self-referral system are described, including the impact on uptake of the intervention as well as participant retention. Because of the potential for recruiting many participants quickly, research teams need to be adequately resourced and the oversight committees prepared to meet at shorter intervals with RCTs of brief interventions.

**Short conclusion:**

Self-referral to a brief intervention for depression with a non-diagnostic title can be a very effective way of recruiting depressed people into trials. However, there are also some challenges.

**Trial registration:**

ISRCTN, ISRCTN26634837. Registered on 10 June 2010.

## Lessons learned

- Using a self-referral system with a ‘non-diagnostic label’ for the intervention can be very successful in recruiting depressed participants.
- Because there can be large numbers of self-referrers, it is important to also monitor the uptake to the intervention and the retention of participants.
- The decision to pause a trial can be very helpful, although hard to take.
- Careful monitoring and problem-solving of the different processes in the trial is required; these processes may include scripting workshop introductions to encourage uptake of the intervention,
- In brief interventions, Trial Management Groups become more important.

## Background

Depression is an important common mental health disorder which is predicted to be the second leading cause of disability by the WHO [1]. However, there is a reluctance to seek help for depression, with only 57% of those with depression seeking help from professionals [2]. The most common reason for not seeking help for depression is low perceived need, and specifically a wish to handle the problems on one’s own [3]. Stigma has been found to be the fourth most common barrier to help-seeking [4].

Randomised controlled trials (RCTs) are the gold standard for assessing the effectiveness of any intervention [5]. However, recruitment to clinical trials for depression is a major problem. An examination of 114 trials funded by two UK agencies (MRC and NIHR Health Technology Assessment (HTA) programmes) conducted between 1994 and 2002 [6] showed that less than a third (31%) reached their original recruitment targets, with over half (53%) needing extensions. In the sequel to that study with 73 trials conducted between 2002 and 2008 [5], a slight improvement was found with 55% recruiting to their original targets and just under half (45%) needing an extension. In the worst-case scenario, recruitment problems have led to some studies being abandoned [7].

There are many individual studies with low recruitment rates. In one study, only 1% were recruited to an Internet-based intervention for depression [8]. In another study of counselling for depression, the trial was abandoned altogether when only one patient was recruited after 5 months, and was redesigned into a qualitative study to learn lessons about why recruitment had been so poor [9]. A large number of depression drug trials have also failed, as occurred with a study of tranylcypromine compared to lamotrigine in bipolar depression when only 29% of targeted patients were recruited [10]. Bower et al. [11] have pointed out that the recruitment problem for trials has serious implications for statistical power, internal and external validity, as well as practical and financial impacts. Recruitment problems also lead to delayed completion of research, thereby reducing timely impacts on patient health and well-being.

Clinicians’ attitudes and patient engagement can be key in recruitment to RCTs. A qualitative systematic review of depression trials [12] concluded that clinicians’ judgments of risk and reward were very significant in whether the clinician mentioned the study to the patient. Three key issues were: firstly, the perceived ‘health state’ of the patient; secondly, clinicians’ attitudes towards research and trial interventions; and, thirdly, engaging the patient. Another study found that GP practitioner attitudes to counselling and research can act as obstacles to recruitment [13]. These include: concern about protecting the vulnerable patient; not feeling confident in asking patients if they may be interested in participating in a research project; and lower priority given to research compared to other clinical and administrative issues.

Patients’ perceptions and misconceptions about the studies and interventions can sometimes also affect recruitment, which is an important aspect for researchers to consider. In a trial of psychological treatment, a large proportion of antidepressant users declined participation [14]. Reasons included previous poor experiences with counselling as well as misunderstandings about the study. In another study, depressed participants reported finding it difficult to participate in the research study, often cancelling appointments at the last minute because of their low mood [15]. On the other hand, depressed users in another study [16] indicated that they felt good about participating, and were interested in an intervention that may help them feel better. Similarly, patients in another study [17] reported enjoying participating in trials.

Finally, another important aspect of recruitment is the method used to recruit. One primary care depression trial for a computerised CBT intervention that recruited ahead of target used both direct referral from GPs and screening of patient details on GP practices’ databases. Direct referral by the GP provided the bulk of participants (72%) compared to database screening [18]. On the other hand, in another study, only a handful of participants were referred by their GPs, with the majority identified through a search of the GP practices’ computerised records [14]. An anxiety study [19] found that over 50% of the participants were recruited though self-referral in response to mail-outs of flyers, whereas physician referral contributed less than 10% of the sample.

The focus of our research for a number of years has been to improve access to brief depression treatments, particularly for those who have not previously sought help [20]. Our aim has been to offer an early intervention to people before their depression becomes chronic. We initially found that a GP referral system to a psychological depression intervention, referred to as ‘depression workshops’, led to a very small number of referrals and had to be abandoned. We then went on to experiment with a self-referral approach, whereby individuals could refer themselves to day-long ‘depression workshops’. We found this yielded more participants [21] but over 90% of these had previously consulted their GPs, were already diagnosed and were already using specialist services. We also found poor attendance at follow-up meetings.

It was only after changing the title from ‘depression workshops’ to ‘self-confidence workshops’ that we found an appreciable increase in recruitment, particularly from people who had not previously sought help [22]. The idea for re-labelling was based on the known close relationship between depression and self-esteem [23]. The term ‘self-confidence’ was used rather than self-esteem because this term is used more often colloquially and we wanted a non-diagnostic ‘lay’ title that was understandable and acceptable to the public. Additionally, self-confidence is a positive term that could give a feeling of hope, a curative factor which is important in therapy [24]. We have found that choosing a ‘sensitively engaging’ title for an intervention could be very important in reaching those who may be reluctant to engage. This is particularly important as the main barrier to help-seeking is not stigma but a low perceived need for treatment [3, 4]. Finally, a key finding from a naturalistic study was that the effects of the day-long self-confidence workshops were not just short-term but were maintained after 2 years, especially for those with depressive symptoms above the threshold [25].

Several discoveries in our previous trials of self-referral further supported this as a valid recruitment method. One discovery was that self-referrers were not just the ‘worried well’ and that 75% had diagnosable problems when psychiatrically interviewed using the CIS-R [26]. Secondly, a proportionally more representative group of the local population from black and ethnic minorities (BME) backgrounds (32%) came forward [22] compared to the GP referral rate of about 25% for the area [27]. This fits in with Gask’s model of access to mental health care [28]; this suggests that, as BME individuals can be reluctant to seek help from GPs [29], offering access to BME groups might not necessarily involve the GP. Thirdly, we found that there were significant proportions of people who had not consulted their GPs but who nevertheless self-referred [26]. This is important given that only about half of those with depression consult their GPs.

In previous studies we had run, all participants who self-referred were offered the intervention, regardless of depressive status. Interventions were also run at a single site.

In the CLASSIC RCT of self-referral to self-confidence workshops, one of our aims was to gather stronger evidence for self-referral using a non-diagnostic title as a feasible recruitment method for depression. We also specifically wanted to assess the proportion of people who would meet criteria for depression from participants who self-referred in this larger study within a multi-site design (eight different sites in south London). Full results of the trial are published in Horrell et al. [30]. In the present paper, we discuss the lessons learned from recruiting using the self-referral recruitment strategy.

## Method

### Design of current study

We descriptively report findings under three main headings:

- Recruitment rates for the 12 months of the project before and after the pause.
- Data regarding attendance at the workshops before and after the pause.
- The follow-up rates before and after the pause.

### Design of original trial

**Fig. 1:**
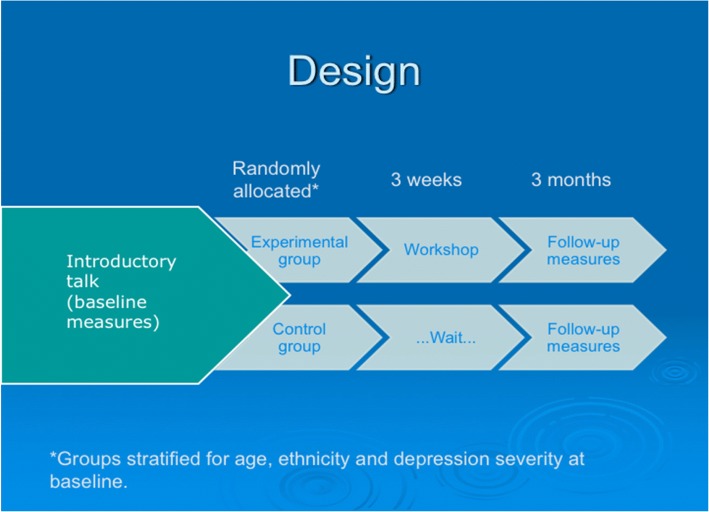
Design of the study

A parallel RCT design was used, with the intervention group compared to a waiting list group (see Fig. 1). The waiting list control design was decided upon to reduce ambivalence about participating in the study [31]. Full details of the CLASSIC trial are reported separately [30].

The study was conducted across eight London boroughs including Lambeth, Greenwich, Lewisham, Croydon, Wandsworth, Bexley, Merton/Sutton and Kingston upon Thames. Workshops were run in non-mental health settings such as leisure centres or libraries in these boroughs as these were considered to be more accessible and acceptable to participants.

Descriptive data about recruitment, uptake of intervention and retention of participants to the trial using a self-referral system have been provided in this paper.

### Measures

The primary outcome measure used was the Beck Depression Inventory—second edition (BDI-II, referred to here as the BDI) [32]. Data about recruitment, attendance at the intervention and attrition were collected systematically.

### Intervention

The intervention was a 1-day CBT workshop about self-confidence and depression, designed to be accessible to members of the general public.

### Method of recruitment

**Fig. 2:**
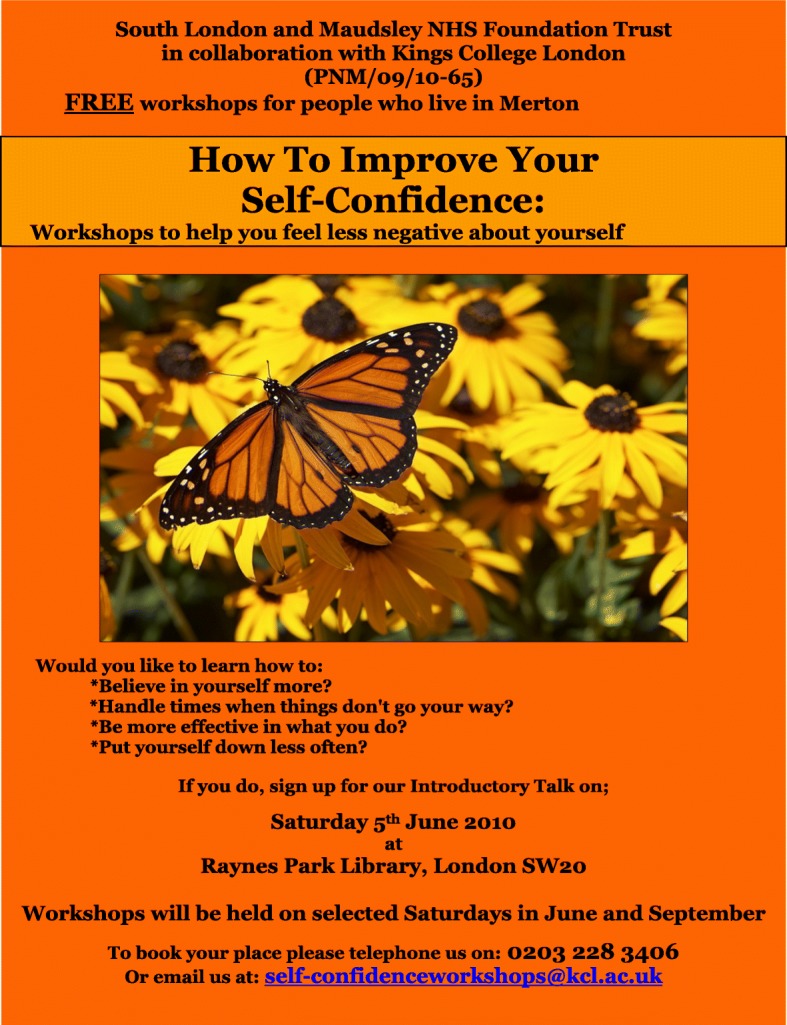
Example of a publicity flyer used for the trial

Participants self-referred by telephone or email following a local publicity campaign to advertise the study, using leaflets and flyers entitled ‘How to improve your self-confidence’. An example is given in Figure 2 which was designed by an advertising consultant to be colourful and striking and, given the possible lack of knowledge about depression and self-esteem, to highlight some of the possible problems that may be helped by the self-confidence workshops.

Interested participants were invited to attend an introductory talk where the study and intervention were outlined. Participants who consented and fitted the inclusion criteria (including scoring above the depression threshold on the BDI) were randomised 1:1 into the intervention or control arm of the trial. Participants were informed of the workshop they were allocated to within the 3-week period after the introductory talks.

### Research and clinical teams

The research team comprised one trial manager and one research worker, with support from an administrator (part-time). We also recruited a team of four part-time research volunteers who offered 1–2 days per week, taking calls and assisting with the workshops. The research team used a single-site research protocol that had been developed for the original pilot study [22] that had been based at one site.

The clinical team comprised four clinical psychologists who formed two separate workshop teams. They used a clinical protocol for the pilot study with one experimental and one control workshop [22].

At month 4, we realised that although recruitment was going very well, there were both lower than expected attendance at the intervention as well as lower than expected follow-up rates. A review meeting with the research team revealed that there were a number of difficulties with this busy trial, including insufficient time for research workers to contact participants for follow-up data, as well as confusion about which participants needed to be followed up. We therefore decided to pause the trial and develop multi-site research protocols and the clinical protocol in months 5–6.

The Trial Steering Group was scheduled to meet 6 months after the start of the study.

## Results

There was initially a relatively large number of depressed self-referrals to the trial (*n* = 135 over 3 months). However, it was observed that attendance at the workshops was poor, with less than 50% attending the allocated day-long workshop (47.4%; *n* = 64). Retention of participants (34.1%; *n* = 46) was also low as we had been aiming to achieve an 80% follow-up rate after 3 months.

We report findings from before and after the pause of the trial when multi-site protocols were developed, under three main headings:

- Recruitment rates for the 12 months of the project before and after the pause.
- Data regarding attendance at the workshops before and after the pause.
- The follow-up rates before and after the pause.

### Recruitment rates

In our previous studies, workshops had been run at one site. The CLASSIC trial was a much more ambitious study with a limited budget aiming to evaluate workshops run in eight different boroughs in south London. The study was also on a tight timeline, with a 21-month study period and a recruitment phase of 12 months (see Table 1).

**Table 1:** Recruitment rates for the 10 sites

Site	Enquiries: telephone or email (*n*)	Introductory talks (*n*)	Ineligible: scoring below 14 on BDI, *n* (%)	Ineligible: not meeting other criteria, *n* (%)	Experimental group (*n*)	Control group (*n*)	Total number (%) recruited and randomised	GP non-consulters, *n* (%)
Lewisham	128	94	22 (23.4%)	23 (24.5%)	26	23	49 (52.1%)	13 (26.5%)
Wandsworth	84	61	13 (21.3%)	0 (0%)	24	24	48 (78.7%)	9 (18.8%)
Merton	68	57	16 (28.1%)	3 (5.3%)	19	19	38 (66.7%)	17 (44.7%)
Total before pause	280	212	51 (24.1%)	26 (12.3%)	69	66	135 (63.7%)	39 (28.9%)
After pause and review of procedures								
Croydon	150	93	15 (16.1%)	5 (5.3%)	36	37	73 (78.5%)	21 (28.8%)
Lambeth	90	58	10 (17.2%)	14 (24.1%)	17	17	34 (58.6%)	9 (27.3%)^a^
Bexley	89	70	8 (11.4%)	11 (15.7%)	26	25	51 (72.9%)	11 (21.6%)
Greenwich	124	85	11 (12.9%)	25 (29.4%)	24	25	49 (57.6%)	8 (16.3%)
Kingston	105	75	13 (17.3%)	17 (22.7%)	22	23	45 (60.0%)	9 (20.0%)
Lewisham 2	92	63	8 (12.7%)	21 (33.3%)	16	18	34 (54.0%)	7 (20.6%)
Croydon 2	112	78	24 (30.8%)	16 (20.5%)	18	20	38 (48.7%)	8 (21.1%)
Total after pause	762	522	89 (17.0%)	109 (20.9%)	159	165	324 (62.1%)	73 (22.5%)
Total	1042	734 (70.4%)	140 (19.1%)	135(18.3%)	228	231	459 (62.5%)	112 (24.5%)^a^

*BDI* Beck Depression Inventory, *GP* general practitioner

^a^One participant withdrew, so data for 458 participants are included, withdrawal was from Lambeth, so percentage for that site was calculated as 9/33 and overall percentage calculated as 112/458

Within the 12-month recruitment period, over 1000 (*n* = 1042) people telephoned or emailed to enquire about attending the workshops. Of these, the majority (734; 70.4.%) came to the introductory talks, with 459 (62.5%) recruited and then randomised (see Table 1). A total of 275 (37.5%) were excluded, with 140 (19.1%) below the clinical threshold, 47 (6.4%) declining and 88 (12.0%) not meeting our other criteria. A total of 382 (83.2%) were followed up. One participant withdrew consent and their data were excluded. Before the pause, the recruitment rate at three sites was 63.7% (*n* = 135) and after the pause, the recruitment rate on seven sites was 62.1% (*n* = 324).

#### Above clinical threshold

At each of our eight sites (see Table 1) we succeeded in recruiting and randomising between 34 and 73 participants who met the inclusion criteria. The average rate of non-depressed self-referrals in our study was 19.1% (range from 11.4 to 30.8% per each intake at each borough).

#### GP non-consulters

Across all eight boroughs, 25% of participants had never previously consulted their GP. For most boroughs, approximately a quarter to a fifth of participants had not consulted their GP. However, nearly half of the participants had not consulted their GPs in Merton (Table 1). The borough with the lowest non-consultation proportion was Greenwich (16.3%).

#### BME groups

We were able to recruit a relatively large proportion of people from BME backgrounds. The proportion of Black participants self-referring was more than 1.5 times that of the local population in five London boroughs and the proportion of Asian participants was more than twice that in the local population in three boroughs [30].

### Low attendance at workshops

Attendance at the experimental workshops was initially lower than expected, with attendance at the first three sites being 38.5%, 58.3% and 63.2% of those recruited at those sites (Table 3).

Anecdotal comments from participants who were recruited suggested they did not always feel sufficiently engaged by the clinical team at the very busy introductory talks when measures were also being taken and therefore did not feel encouraged to attend the intervention workshop. This led to a higher than expected rate of non-attendance.

There were also problems in letting participants know whether or not they had been randomised to the experimental or control group workshops. Much of the difficulty was that the participants were only randomised after they had completed their baseline assessments at the introductory talks but these were not always fully completed. Telephone calls therefore had to be made to participants after the introductory talks to check on the answers, and if they were not available there was a further delay. The result of this was that sometimes participants were not informed about the workshop they had been allocated to until the last minute, by which time they may have made other arrangements.

When we realised that there were problems, our strategy was to pause recruitment for 2 months (months 4–5) while the procedures were reviewed and new procedures were put into place to improve attendance, rather than continue running the trial whilst attempting to make the changes. In addition to the changes in the single-site protocols, staff were also given training about systematically collecting all baseline data and collecting outcome data for the intention-to-treat (ITT) analysis. Table 2 presents the revised multi-site protocol to improve attendance at the workshops.

**Table 2:** Revised protocol to improve attendance at workshop

(a) Workshop leaders leading the introductory talks were asked to increase participants’ hope of improvement as a result of attending the workshop programme (b) Workshop leaders at the talks were asked to emphasise the importance of answering all questions, or else there would be a delay in letters being sent out about the workshops (c) Additional research volunteers were recruited and sent to introductory talks and asked to check baseline data quality on the day itself (d) To maintain engagement, research workers were asked to email/telephone within 1 week after the introductory talk or immediately after randomisation to inform participants about the workshop to which they had been allocated	

The new multi-site protocol proved to be very effective (see Table 3). The attendance at the intervention improved from 38.5% at the first site to 94.4% at the last site. The attendance rate for the experimental groups at the first three sites was 50.7%, whereas the rate for the other seven sites was 72.3%. Interestingly, the attendance rate for the control groups also improved from 42.4% to 64.2%. The lower increase may be due to the 3-month delay before the intervention was offered. The overall improvement for both groups increased from 47.4% to 68.2%.

**Table 3:** Intervention attendance rates before and after the pause and timing of the workshops at 10 sites

			Attendance rate		
Site	Date of workshop	Trial month	Number of experimental group invited (attended)	Number of control group invited (attended)	Total
Lewisham	April 2010	1	26 (10) = 38.5%	23 (11) = 47.8%	
Wandsworth	May 2010	2	24 (14) = 58.3%	24 (10) = 41.7%	
Merton	June 2010	3	19 (12) = 63.2%	19 (7) = 36.8%	
Average before pause			69 (36) =50.7%	66 (28) = 2.4%	135 (64) = 47.4%
After pause and review of procedures					
Croydon	September 2010	6	36 (19) = 52.8%	37 (27) = 73.0%	
Lambeth	October 2010	7	17 (13) = 76.5%	17 (8) = 47.1%	
Bexley	November 2010	8	26 (16) = 61.5%	25 (17) = 68.0%	
Greenwich	January 2011	10	24 (20) = 83.3%	25 (15) = 60.0%	
Kingston	February 2011	11	22 (17) = 77.3%	23 (15) = 65.2%	
Lewisham 2	March 2011	12	16 (13) = 81.3%	18 (9) = 50.0%	
Croydon 2	March 2011	12	18 (17) = 94.4%	20 (15) = 75.0%	
Average after pause			159 (115) = 72.3%	165 (106) =64.2%	324 (221) = 68.2%
Overall			228 (151) = 65.8%	231 (134) = 58.0%	459 (285) = 62.1%

### Low retention in study

At month 4 in the recruitment phase, the research team had noticed low outcome data follow-up rates from the first three sites. The data were then systematically checked by the CTU who found that while the recruitment rate was initially well above target, attrition at the follow-up stage was far higher than the 15% allowed for in the original sample size calculation. By month 4, the attrition rate was approximately 65–70%. At this point, the study was at risk of being underpowered, due to the large proportion of missing outcome data at follow-up.

More specifically, we discovered the following:

- The single-site protocol that was being used did not set out specific enough data collection timelines, so that 3-month follow-up data collection was being delayed until later.
- The original single-site protocol did not explicitly state that data had to be collected for all randomised participants and did not adequately explain the distinction between failure to participate in the randomised intervention and actual withdrawal from data collection.

The single-site protocol was therefore revised to reduce attrition (see Table 4). The new multi-site protocol explicitly stated that outcome data must be sought from all participants who had not withdrawn from the study and that outcome data should be collected at or very soon after the 3-month time period.

**Table 4:** Revised protocol to improve retention

(a) Workshop leaders and research staff were asked remind participants about the importance of the booster and follow-up at the workshop, by letter or email, immediately after the workshops and 1 month before follow-up (b) Research staff were asked to remind control workshop participants about their allocated workshop after the introductory talk, as well as 1 month and 1 week before the workshop (c) Research staff were asked to strengthen questionnaire-chasing letters (d) Research staff were also asked to follow-up experimental participants by telephone, letter or email who had not attended their allocated workshop (e) Research staff were asked to follow-up controls by telephone, letter or email any time after the 3-month period, as these participants could attend any control workshop that was being run (f) A script for following-up participants was developed which was more focused (g) A set of procedures was developed for following up participants, which included: (i) a pack of outcome assessment forms and letters (ii) a schedule for five instances of telephone contact with the participant (iii) if this failed, a shortened data assessment pack (by email or post)	

Additionally, the sample size was increased from the initial number of 320 over eight sites (40 per site) [30] to 420 over 10 sites (42 per site). This new sample size was met by enrolling extra sites.

The new multi-site protocol proved to be very effective (see Table 5). The new attrition rate ranged from 5 to 18% across sites. It was recognised that the original estimate of 15% was probably rather ambitious as the actual overall attrition rate was 16.8% across the whole trial.

**Table 5:** Attrition rates for the 10 sites before and after the pause

	Total number (%) recruited and randomised	3-month follow-up before protocol change, *n* (%)	3-month follow-up after protocol change, *n* (%)
Lewisham	49 (52.1%)	17 (34.7%)	33 (67.3%)
Wandsworth	48 (78.7%)	17 (35.4%)	35 (72.9%)
Merton	38 (66.7%)	12 (31.6%)	33 (86.8%)
Sub-total for three sites	135	46 (34.1%)	101 (74.8%)
After pause and review of procedures			
Croydon	73 (78.5%)		64 (87.7%)
Lambeth	34 (58.6%)		28 (82.4%)
Bexley	51 (72.9%)		42 (82.4%)
Greenwich	49 (57.6%)		39 (79.6%)
Kingston	45 (60.0%)		42 (93.3%)
Lewisham 2	34 (54.0%)		30 (88.2%)
Croydon 2	38 (48.7%)		36 (94.7%)
Sub-total for seven sites	324		281 (86.7%)
Total	459 (62.5%)		382^a^ (83.2%)

^a^One additional person withdrew consent and one additional person provided incomplete follow-up BDI data

## Discussion

### Recruitment rate

We managed to recruit a very large number of depressed participants (*n* = 459) into our trial using the self-referral method within a very short space of time (12 months) across multiple sites. This represented 62.5% of the 734 people who came to the introductory talks about the workshops.

Compared to other studies, this was a very good recruitment rate. When comparing with larger depression trials which did successfully recruit, the larger REEACT study randomised 691 patients from 83 out of 100 GP practices to a computerised treatment over a period of 20 months [33]. The cut-off values used were on the PHQ-9 and the ICD-10. This gave a recruitment rate of 35 patients per month. In another large study, the COBALT trial, 469 patients with treatment-resistant depression (defined as on medication for at least 6 weeks, BDI scores ≥ 14 and ICD-10 diagnosis of depression) were recruited from 73 UK general practices over 23 months [34]. Treatment was additional CBT to medication. This gave a recruitment rate of 20 patients per month. In comparison, we recruited 459 participants to a psychological treatment with a non-diagnostic label of ‘self-confidence’ from eight areas within a 12-month period, giving an average monthly rate of 38. The CLASSIC trial recruitment rate was similar to or faster than both the larger REEACT and COBALT trials.

This supports the use of a self-referral system, in combination with non-diagnostic labelling of the intervention, to recruit depressed participants.

### Value of self-referral

#### Clinical threshold

We might have expected a large proportion of people recruited by self-referral not to meet the threshold for depression on the BDI. On average, only approximately 20% did not meet the clinical threshold. This was not appreciably different from those reported in other studies, although precise figures from other studies are not always reported. In a trial of telephone-administered CBT for anxiety where flyers were sent to older adults living in rural areas in the USA, it was reported that 32.3% of those screened did not have Generalised Anxiety Disorder (GAD) [19]. In a study of computerised CBT, 18.4% did not meet the inclusion criteria although more precise figures enumerating reasons for rejection were not given [18].

#### BME groups

We found that this method of recruitment led to better access for BME groups [20]. More detail is given in the original paper [30]. This is consistent with findings that BME groups were more likely to self-refer than to be referred by a GP to a psychological therapy service in the UK (IAPT service) [27, 35]. We also found that those of black, Asian or other ethnic background were more likely to be GP non-consulters than white or mixed ethnic groups (*p* = 0.003). While 19.8% (62/313) of white participants were GP non-consulters, 35.8% (24/67) of black participants, 39.6% (19/48) of Asian participants, 18.2% (4/22) of mixed participants and 37.5% (3/8) participants of ‘other’ ethnicities had not previously consulted a GP about their psychological problems.

#### GP non-consulters

The self-referral system engages people who have not previously consulted their GPs [20]. Across all eight boroughs, 25% of participants had never previously consulted their GP. This is consistent with previous studies where we found that the self-confidence workshops, open to members of the public and not just for depressed participants, attracted 39% who had not previously sought help [22]. Given the low rate of help-seeking for depression [2], this suggests that self-referral could provide an important means of access to greater numbers of depressed individuals.

### Why did self-referral work?

One obvious answer as to why self-referral worked is that the self-referral system circumvented the GP system as participants could refer themselves. It could be advantageous to allow depressed individuals to sidestep the problem of busy GPs or other clinicians who might struggle to find time to focus on research [13]. It thus avoids issues to do with protecting the patient’s vulnerable ‘health state’ [12] or ‘concern about protecting the vulnerable patient’ [13]. So, patients with depressive symptoms could refer themselves even if the GP does not refer. The downside may be that patients scoring below the clinical threshold may refer themselves unnecessarily to these interventions. However, it is important to remember that people with depression do tend to under-refer [2] and that people who self-refer do not tend to be the ‘worried well’ but are likely to have diagnosable problems [26]. Finally, with this system, we also avoided the ethical problems of GPs or other clinical staff at the GP practice acting as gatekeepers for researchers because we were not relying on referrals from GPs.

Secondly, a related aspect is the relatively low percentage (50%) of people who go to their GPs if they are depressed [2]. A self-referral system can therefore also reach more people who are depressed but who do not consult their GPs. For example, some BME groups will avoid going to the GP if they have mental health problems because they may not perceive it as a medical problem [29]. This fits in with the model proposed by Gask et al. [28], where bypassing referral at the GP level, relying more on community engagement and using psychosocial interventions can increase access for difficult to engage groups such as BME persons.

Thirdly, we believe that we successfully engaged participants. The use of flyers and non-diagnostic labelling of the intervention as self-confidence rather than depression workshops was likely to be very important [22]. Despite this non-diagnostic title, we found a surprisingly large proportion of people who had scores above our clinical depression threshold. In a previous study, we found that 73% of self-referrers to self-confidence workshops met ICD-10 clinical thresholds [26]. We have gone on to use similar non-diagnostic labels for workshops for other health problems with success, for example for stress rather than anxiety, and for sleeping problems rather than insomnia [36].

### Waiting list design

The waiting list design also meant that participants would eventually receive the treatment being evaluated. This therefore reduced the ambivalence problems of ‘attitudes towards research and trial interventions’, particularly randomisation, when patients may be allocated to the control group and never receive the treatment being trialled [12, 31]. Other studies [17] also reported participants wanting to be altruistic but also not wanting to risk getting worse and wanting something in return. However, the disadvantage of the waiting list control for the researcher is that longer-term follow-up of both experimental and control groups is difficult, because the control group will always receive the treatment. Only long-term follow-up of experimental group participants will be possible in such a design, therefore not allowing for randomised comparisons.

### Need for revised multi-site protocols

The unexpectedly rapid recruitment rate led to us being the victims of our own success as it meant that some tasks were compromised; most importantly, gathering of outcome data at follow-up. These necessitated a considered revision of the trial processes so that we could both successfully recruit large numbers, encourage attendance at the intervention and then follow them up consistently.

At the time of the study in 2009, no formal study protocol for trials of psychological therapies was required by the funders or sponsors. The PI, trial manager, research workers and therapists therefore relied on the single-site protocols and verbal communications between team members. Sufficiently detailed multi-site trial protocols were therefore developed during the ‘pause’, which made it clearer to research workers which (e.g. follow-up assessments) and when additional tasks for the trial across the different sites needed to be done. Clinical trials are now required (and have been for some time) to have a study protocol for the trial that is submitted to the ethics committee and sponsor, and used by the study team in the conduct of the trial. The ‘Standard Protocol Items: Recommendations for Interventional Trials’ (SPIRIT) provide guidance on writing protocols [37–40].

The procedures we adopted helped us to follow-up 382 (83%) of those recruited, which is reasonable for a trial of a mental health intervention, particularly for one where participants were recruited from the community. Increasing the sample size to counter the high attrition rate helped us ensure we had a sufficiently powered trial.

### Resources needed

We would also like to mention two other factors. Firstly, we refer to the resources needed to run a trial of this size. Because of the upper financial limit stipulated by the funding stream we had applied for, we had asked for two research workers. With hindsight, three research workers were really needed given the work involved in running a trial of this size.

Secondly, in order for a self-referral system to work in a large study, time was needed for setting up the self-referral system in each of the eight sites as well as promoting the workshops [41]. Tasks included identifying possible community sites accessible by public transport and ensuring there were sufficiently large rooms for the workshop. The exact sites at which each of the eight workshops was going to take place had to be decided early, before the advertising material could be finalised. There also needed to be a response time for participants to be able to see the publicity material and to act on this. In the early days of the trial, the amount of time needed to set up took longer than anticipated. It sometimes took 3 weeks to find a suitable location rather than the anticipated 1–2 weeks. Once the publicity material was circulated, we then had a large number of people telephoning or emailing enquiring about the workshops. This had a knock-on effect on the time needed for collecting outcome measures for the follow-up.

For this trial, we decided to recruit four part-time voluntary assistant psychologists to help the research workers manage telephone calls and emails from people interested in participating. This meant the voluntary staff required training in research processes, and in interacting effectively with members of the public on the telephone and at the workshop events. They also had to record and coordinate detailed information, such as whether or not potential participants could come on both days for the workshop so that they could be randomised. They learned a great deal from their experience but there was a cost to us in terms of training and ensuring that procedures were followed.

### CTU and oversight committees

The other major lesson we learned was to do with the role of the CTU and oversight committees. The role of the CTU was key to the success of this trial in helping to examine the low follow-up rates early on, and then jointly rectifying the problem before it became too serious. However, in trials with a brief intervention and a relatively short follow-up period (12 weeks in this case), data checks need to be organised earlier than normal. The original post-recruitment Trial Steering Committee (TSC) meeting was planned for 6 months into a 12-month recruitment period. Attrition problems would have been too late to resolve at that stage. While the TSC meetings are likely to be less frequent, Trial Management Groups (TMG) could play a key role in brief trials as these tend to meet more often to review recruitment attrition and adherence to intervention. This is particularly relevant with brief trials of shorter, more accessible interventions such as those motivated by the Improving Access to Psychological Therapies (IAPT) Programme.

## Overall conclusions

Issues with recruitment are crucial to trial success, and should be tackled as early as possible. We have shown that self-referral can be a very important strategy in increasing the number of people recruited with depression. We would certainly encourage researchers to consider self-referral for depression trials where appropriate. Self-referral could also be a useful adjunct to the clinician referral route. Putting procedures in place to handle large numbers of self-referrers and ensure robust outcome data gathering are needed to ensure the success and utility of such trials.

Important lessons were to ensure that participants, once recruited, were engaged clinically and followed up as completely as possible. With this very busy trial, it was challenging to meet the important aim of keeping participants engaged in the intervention, as well as in follow-up. The fact that we attracted so many people meant that we initially lost sight of procedures that were needed for successfully following up participants. A revision of the procedures described in the original single-site protocols and investment in research staff training remedied this oversight in a timely manner. Generally, we think we succeeded in ‘engaging the patient’ initially, but needed changes to the original single-site protocols to ensure we engaged them right through to follow-up across all of the sites.
